# Toll-like receptor 2 confers partial neuroprotection during prion disease

**DOI:** 10.1371/journal.pone.0208559

**Published:** 2018-12-31

**Authors:** James A. Carroll, Brent Race, Katie Williams, Bruce Chesebro

**Affiliations:** Laboratory of Persistent Viral Diseases, Rocky Mountain Laboratories, National Institute of Allergy and Infectious Diseases, National Institutes of Health, Hamilton, MT, United States of America; Istituto di Ricerche Farmacologiche Mario Negri IRCCS, ITALY

## Abstract

Neuroinflammation and neurodegeneration are common during prion infection, but the mechanisms that underlie these pathological features are not well understood. Several components of innate immunity, such as Toll-like receptor (TLR) 4 and Complement C1q, have been shown to influence prion disease. To identify additional components of innate immunity that might impact prion disease within the central nervous system (CNS), we screened RNA from brains of pre-clinical and clinical 22L-infected mice for alterations in genes associated with innate immunity. Transcription of several genes encoding damage-associated molecular pattern (DAMP) proteins and receptors were increased in the brains of prion-infected mice. To investigate the role of some of these proteins in prion disease of the CNS, we infected mice deficient in DAMP receptor genes *Tlr2*, *C3ar1*, and *C5ar1* with 22L scrapie. Elimination of TLR2 accelerated disease by a median of 10 days, while lack of C3aR1 or C5aR1 had no effect on disease tempo. Histopathologically, all knockout mouse strains tested were similar to infected control mice in gliosis, vacuolation, and PrPSc deposition. Analysis of proinflammatory markers in the brains of infected knockout mice indicated only a few alterations in gene expression suggesting that C5aR1 and TLR2 signaling did not act synergistically in the brains of prion-infected mice. These results indicate that signaling through TLR2 confers partial neuroprotection during prion infection.

## Introduction

Prion diseases are fatal infections of the nervous system caused by the misfolding of the normal host protein (PrPc) into a self-propagating infectious conformer (PrPSc or PrPres) [1–3]. Prion disease results in gliosis, neuronal damage, and neuroinflammation [3–7], and proinflammatory cytokines and chemokines produced by microglia, astroglia, and other cell types in the brain increase as the disease progresses [6]. It remains unclear whether this neuroinflammation is beneficial or harmful to the prion-infected individual, but it has been proposed that microglia instigate the majority of the neuroinflammatory response [8].

Microglia serve many functions in the healthy CNS and are considered the first line of defense responding to infection and damaged tissue [9–13]. These glial cells constantly survey their microenvironment, using a cadre of receptors on their surface recognizing pathogen-associated molecular patterns (PAMPs) and damage-associated molecular patterns (DAMPs) to detect abnormalities such as infection or damage [13–16]. PAMPs include exogenous molecules derived from microorganisms (such as LPS, unmethylated CpG, or diacylated lipoproteins) that allow the host to distinguish “self” from “non-self”, while DAMPs include endogenous proteins, lipids, and nucleic acids released by damaged or dead cells [17–20]. These exogenous and endogenous signals, though of differing origins, often utilize overlapping recognition receptors to elicit an inflammatory response.

Recently, we identified microglia as critical in host defense during prion infection in the CNS [21]. Mice treated with the potent CSF-1R inhibitor PLX5622 to eliminate microglia are more susceptible to prion disease and succumb to disease 20 to 30 days faster than untreated mice. Moreover, PLX5622-treated mice accumulate PrPSc earlier and to a greater extent throughout the disease course. Thus, the neuroprotective effects of microglia might be due to microglial phagocytosis and clearance of PrPSc, resulting in extended survival. Signaling through DAMP receptors that are predominately expressed on the surface of microglia may contribute to this neuroprotective function.

There is ample evidence that the complement system is involved in extra-neural prion infection. Complement component C1q is essential for neuroinvasion when mice are inoculated intraperitoneally (ip) with prions, and prion interaction with C1q is required for follicular dendritic cell infection and transmission in the spleen [22]. Furthermore, elimination of component C3 or its receptor Cr2 delays neuroinvasion after ip inoculation [22–24]. In addition, mice deficient in complement receptors CD21/35 are partially resistant to terminal prion disease when infected ip [25,26]. Interestingly, a study using C5-deficient mice, which is a component of the complement membrane attack complex that disrupts targeted cell membranes, suggested that complement component C5 was not required for prion pathogenesis when mice were challenged either by intracerebral (ic) or ip routes [27].

Other experiments implicate Toll-like receptor (TLR) involvement in prion disease. Prion disease progression was delayed in mice injected with complete Freund’s adjuvant [28]. Conversely, preexposure of infected mice to LPS [29] or Poly[I:C] [30] worsened prion pathology. Immunization with unmethylated CpG DNA delayed prion disease [31], but these findings are controversial and possibly due to damage of peripheral lymphoid structures [32] and not a direct result of TLR9 signaling. Further studies in C3H/HeJ mice that have a constitutive defect in TLR4 signaling indicate TLR4 is neuroprotective, as C3H/HeJ mice reach clinical end-point faster than C3H/HeOuJ control mice when infected ic or ip with scrapie prions [33]. These results imply that selective TLR signaling may enhance a protective response in prion-infected mice and demonstrate that prion pathogenesis can be altered by host innate immune responses.

In the present paper, we screened for changes in expression of genes encoding DAMP-proteins and receptors in the brains of mice infected with the mouse-adapted scrapie strain 22L to identify potential innate immune pathways involved in prion disease. We identified increases in transcripts encoding DAMP receptors TLR2, C3aR1, and C5aR1 that are predominantly expressed by microglia [34]. Mice deficient in expression of these genes were then infected with prions and compared to infection in wild-type controls. Our results indicated that signaling through TLR2 prolonged survival, but deletion of *C3aR1* and *C5aR1* had no effect on prion infection.

## Materials and methods

### Ethics statement

All mice were housed at the Rocky Mountain Laboratories (RML) in an AAALAC-accredited facility in compliance with guidelines provided by the Guide for the Care and Use of Laboratory Animals (Institute for Laboratory Animal Research Council). Experimentation followed RML Animal Care and Use Committee approved protocol 2015-026E.

### Mice and scrapie inoculations

*Tlr2*^*-/-*^ (Jax# 004650), *C3aR1*^*-/-*^ (Jax# 005712), *C5aR1*^*-/-*^ (Jax# 006845) and BALB/cJ (Jax# 000651) mice were purchased from The Jackson Laboratory. C57BL/6 and C57BL/10 were obtained from colonies maintained at Rocky Mountain Laboratories. All mice were housed 3 to 4 animals per cage.

For scrapie inoculation, mice were anesthetized with isoflurane and inoculated ic. For the initial screening of changes in expression of genes encoding innate immune and DAMP proteins, C57BL/10 mice at 4–6 weeks of age were inoculated via the left and right hemispheres with 20 μl per hemisphere of a 1.0% w/v 22L scrapie brain homogenate stock (final of 8.0 x 10^5^ LD50) or 1.0% w/v normal brain homogenate stock (mock control) in phosphate buffered balanced saline with 2% fetal bovine serum. Following infection, mice were euthanized at a pre-clinical time point (94 dpi) or monitored for onset of scrapie signs. Six to eight mice were euthanized at 94 (pre-clinical) and 131 (clinical) dpi and brains were collected.

For survival curve studies using *C3aR1*^*-/-*^, *C5aR1*^*-/-*^, and Balb/c mice, groups were inoculated ic in the left hemisphere at 4–6 weeks of age with our standard inoculum of 30 μl of a 1.0% w/v 22L scrapie brain homogenate stock, therefore each mouse received a dose of 6.0 x 10^5^ LD50. In contrast, groups of 4–6 weeks old *Tlr2*^*-/-*^ and C57BL/6 mice were inoculated ic in the left hemisphere with 30 μl of a 0.01% w/v 22L scrapie brain homogenate stock, thus each mouse in this experiment received a dose of 6.0 x 10^3^ LD50. This lower LD50 was shown to be adequate in studies performed with C3H/HeJ mice to evaluate TLR4 in prion pathogenesis [33], and using this infectious dose allowed for better comparison between studies.

Mice were observed 2–3 times per week by observers blinded to the mouse genotype. Mice were euthanized when they reached an advanced stage of scrapie disease, characterized by an increased degree of somnolence, reluctance to move, muscle weakness (kyphosis, wasting), clear gait abnormalities, poor grooming habits, and an overall decrease in body condition. Brain tissue was dissected for future use in histology, western blot, or RNA gene expression assays. Survival curves were created using GraphPad Prism version 7, and statistical analysis was performed on the curves by Mantel-Cox log rank.

### Preparation of brain homogenates for protein analysis from mice

Brains were homogenized (20% w/v) using a Mini Bead Beater (BioSpec Products) as previously described [6,35] in ice-cold Cell Lysis Buffer (Bio-Rad) supplemented with 2 X Complete EDTA-free protease inhibitor cocktail (Roche) and 1 X cell lysis factors 1 and 2 (Bio-Rad) consisting of sodium orthovanadate and sodium fluoride to prevent dephosphorylation of proteins. Homogenates were stored at -80°C until use.

### Quantitation of the anaphylatoxins C3a, C4a, and C5a

Enzyme-linked immunosorbent-assays (ELISA) for the quantitation of complement fragments C3a (catalog # 024347), C4a (catalog #024352), and C5a (catalog # 024362) were purchased from US Biologicals Life Sciences. All reagents were provided in the ELISA kits. Briefly, 100 μl of 10% brain homogenate (C4a and C5a) or 100 μl of 5.0% brain homogenate (C3a) was added to the well and incubated for 2 hours at 37°C. The liquid was removed and 100 μl of detection reagent A was added to the well and incubated for 1 hour at 37°C. Wells were aspirated and washed 3 times for 2 min each with 350 μl wash solution. The wash was removed and 100 μl of detection reagent B was added to the well and incubated for 30 minutes at 37°C. Wells were aspirated and washed 5 more times with wash solution. The wash was once again removed and 90 μl of 3,3′,5,5′-tetramethylbenzidine substrate was added to the well for 15 to 20 minutes. To stop the reaction, 50 μl of stop solution was added to the well. Plates were then read at 450 nm. Concentrations of bound C3a, C4a, or C5a were estimated by linear regression (GraphPad Prism version 7) using controls provided with the kit that were serially diluted 2-fold. Ranges of detection for C3a and C5a were 15.6–1000 pg/ml. Ranges for C4a were 31.2–2000 pg/ml. Determined concentrations in pg/ml of diluted brain homogenate were converted to pg/mg of brain tissue for comparison and presentation. Statistical analysis was performed by one-way analysis of variance (ANOVA) using GraphPad Prism version 7.

### RNA isolation

Mouse brain halves were homogenized in 3.0 ml ZR RNA Buffer (Zymo Research) and stored up to 5 days at -80°C before processing. Total RNA was isolated using the Quick-RNA MidiPrep (Zymo Research) and treated with 4 Units of DNase I (Ambion) for 1 hour at 37°C. High-quality RNA was purified using the RNA Clean & Concentrator-100 (Zymo Research) per manufacturer’s instructions and eluted from the columns in 300–400 μl RNase-free water. For RNA isolation from the thalamus of 22L-infected or uninfected mice, thalamus tissue was excised and homogenized in 1.0 ml ZR RNA Buffer (Zymo Research) and stored up to 5 days at -80°C before processing. Total RNA was isolated using the Quick-RNAMiniPrep (Zymo Research), eluted with 75 ul nuclease-free water with 1 x RNase Inhibitor (SUPERase-In, Ambion). All RNA was stored at -80°C until use.

### qRT-PCR analysis

For quantitative analysis of changes in transcription of genes encoding Complement and DAMP proteins and receptors, we compiled data using multiple qRT-PCR arrays available from Qiagen (Mouse G Protein Coupled Receptors, PAMM-3009ZE, Mouse Wound Healing PAMM-121ZE, Mouse Innate & Adaptive Immune Responses PAMM-052ZE, and Mouse Aging PAMM-178ZE). For quantitative analysis of changes in transcription of proinflammatory genes, we employed the Mouse Inflammatory Cytokine & Receptors PAMM-011ZE qRT-PCR pathway-focused array. For each array, 400 nanograms of high-quality RNA from each sample was reversed transcribed to synthesize cDNA using the RT^2^ First Stand Kit per manufacturer’s instructions (Qiagen). Each cDNA reaction was mixed with 2x RT^2^ SYBR Green Mastermix purchased from Qiagen with RNase-free water to a final volume of 1.3 ml. Ten microliters was then added to the appropriate well of a 384-well format plate. The analysis was carried out on an Applied Biosystems ViiA 7 Real-Time PCR System with a 384-well block using the following conditions: 1 cycle at 10 min, 95°C; 40 cycles at 15 s, 95°C then 1 min, 60°C with fluorescence data collection. Melting curves were generated at the end of the completed run to determine the quality of the reaction products. Raw threshold cycle (Ct) data was collected with a Ct of 35 as the cutoff. Ct data was analyzed using the web-based RT^2^ Profiler PCR Array Data Analysis (https://www.qiagen.com/us/shop/genes-and-pathways/data-analysis-center-overview-page/). All Ct values were normalized to the average of the Ct values for the housekeeping genes *Actb*, *Gapdh*, and *Hsp90ab1*. Changes in transcription were calculated by the software using the ΔΔCt based method. Statistical analysis was performed by unpaired Student’s t-test on the mean ΔCt values for each gene in the control and experimental groups, with a greater than 2-fold change and with p value of ≤ 0.05 considered significant. Each group consisted of a minimum of 3 or more independent RNA samples.

### Immunoblotting for PrPres

For PrPres immunoblotting, tissue samples were analyzed as described previously [21,36,37]. Briefly, 0.18 or 0.36 mg of whole brain equivalents were treated with proteinase K, separated by SDS-PAGE, transferred to polyvinylidene difluoride, and probed with a 1:100 dilution of monoclonal human anti-PrP antibody D13 (kindly provided by R. Anthony Williamson). The secondary antibody was peroxidase-conjugated anti-human IgG used at 1:5,000 (Sigma), and immunoreactive bands were visualized using an enhanced chemiluminescence (ECL) detection system (GE Healthcare). Densitometry on unsaturated immunoreactive bands was performed on exposed film using the Bio-Rad ChemiDoc MP system. Adjusted volumes for immunoreactive bands were calculated by taking the total band volume and subtracting the global background using Image Lab software version 5.0 (Bio-Rad). Statistical analysis of densitometry by two-tailed Student t-test was performed using GraphPad Prism version 7.

### Immunohistochemistry

Mice were euthanized, brains were removed, and half of the brain was placed in 3.7% phosphate buffered formalin for 3 to 5 days before dehydration and embedding in paraffin. Serial 5-μm sections were cut using a standard Leica microtome, placed on positively charged glass slides, and dried overnight at 56°C. Slides were stained with a standard protocol of hematoxylin and eosin (H&E) for observation of overall pathology. For the detection of microglia, sections were probed with antibodies against ionized calcium-binding adapter-1 (IBA-1). For astrocytes, sections were probed with antibodies to glial fibrillary acidic protein (GFAP). Slide processing was completed in a Discovery XT slide stainer (Ventana, Tucson, AZ) and read by microscopy as previously described [36,38].

## Results

### Initial screen for alterations in expression of DAMP/innate immune genes in 22L-infected C57BL/10 mice

In the present paper we used several PCR arrays with an expanded panel of genes to screen for changes in expression of DAMP/innate immune genes at pre-clinical (94 dpi) and clinical (131 dpi) times during 22L scrapie infection. Initially, we assayed RNA isolated from the thalamus at 60 dpi, since this region is an early site of pathology and agent replication. Several genes associated with the complement system (C4b, C1qa, C1qb, and C1qc) were increased in the thalamus at 60 dpi, and these genes were also increased in whole brain samples taken at later timepoints (Table 1). The complement-associated gene, *C4b* (C4 isoform B or C4 serum substance (*C4Ss*)), showed the highest fold-change in the thalamus and the brain. The increase in all the genes of the complement system reported herein have been detected during infection by other prion strains, including RML, 301V, and ME7 [39–42]. Thus, these changes are not specific to a single prion strain type.

**Table 1:** Screen for temporal changes in gene expression associated with the complement cascade and activation during infection with scrapie strain 22L.

Gene	Thalamus 60 dpi		Brain 94 dpi		Brain 131 dpi		Complement component:
	FC	P value	FC	P value	FC	P value	
*C4b* (isoform B or *C4Ss*)	**2.9**	****	**8.3**	***	**16.4**	****	C4B (serum substance)
*C3*	-1.1	Ns	**2.2**	**	**13.6**	**	C3
*C1qa*	**2.6**	****	**3.5**	***	**11.5**	****	C1, q subcomponent, alpha polypeptide
*C1qb*	**2.7**	****	**3.6**	**	**10.8**	****	C1, q subcomponent, beta polypeptide
*C1qc*	**2.2**	****	**2.9**	***	**7.1**	****	C1, q subcomponent, C chain
*C1s1*	1.2	ns	1.6	ns	2.7	ns	C1, s subcomponent
*C4a* (isoform A or *C4Slp*)	1.4	ns	-1.2	ns	-1.4	ns	C4A (sex-limited protein)
*Cfh*	1.7	ns	1.5	ns	1.9	ns	factor h
*Cfhr1*	1.3	ns	1.6	ns	-1.1	ns	factor H-related 1
*Clu*	1.2	ns	1.2	ns	1.5	ns	Clusterin (Apolipoprotein J)
*Crp*	ND		1.1	ns	-2.0	ns	C-reactive protein, pentraxin-related
*Mbl2*	ND		1.3	ns	2.0	ns	Mannose-binding lectin (protein C) 2
*Vtn*	ND		1.1	ns	1.1	ns	Vitronectin

Fold Change (FC) in 22L C57BL/10 infected mice relative to uninfected C57BL/10 mice (n = 3 for all cohorts).

ND = not determined

P values are calculated using the student t-test

* P value ≤ 0.05

** P value ≤ 0.01

*** P value ≤ 0.001

**** P value ≤ 0.0001, ns = not significant.

In contrast, during scrapie infection we saw no increase in expression of regulators of the complement cascade (Table 1, lower section). For example, genes encoding the complement regulators factor H (*Cfh*) and factor H-related protein 1 (*Cfhr1*), which influence the alternate pathway of complement activation by accelerating convertase decay [43], were unchanged. Furthermore, genes encoding the proteins Clusterin (*Clu*) and Vitronectin (*Vtn*) that prevent assembly of the complement membrane attack complex [43] were also unchanged. However, components of the complement cascade were increased early in the brain during 22L infection, thus complement activation in the brain may progress without a concomitant increase in regulatory mechanisms during prion disease.

During scrapie infection, we also identified increased expression of DAMP receptor genes in the brain (Table 2). Some of these alterations (i.e. *Tlr2*, *Ddx58*, and *C3ar1*) have been previously identified [39,42,44], but other changes reported here, such as *Adora3*, *C5ar1*, *Tlr1* and *Tlr8*, have not been detected previously. Notably, *Tlr1* and *Tlr2* were significantly increased by 60 dpi in the thalamus, and *Tlr2* had the highest fold change at clinical end-point (Table 2). Most of the increased DAMP-associated genes are likely to have been produced by microglia as they are predominantly expressed by microglia [34] in normal healthy brain (Table 2, underlined genes).

**Table 2:** Screen for temporal changes in gene expression of damage-associated molecular pattern (DAMP) recognition receptors and associated proteins during infection with scrapie strain 22L.

**Changed**								
**Gene**	**Thalamus 60 dpi**		**Brain 94 dpi**		**Brain 131 dpi**		**Description**	**Effect of loss on prion disease**
	**FC**	**P value**	**FC**	**P value**	**FC**	**P value**		
*Tlr2*	**2.5**	***	**5.1**	****	**20.6**	****	Toll-like receptor 2	Herein
*Olr1 (Lox-1)*	**ND**	**-**	**7.9**	****	**18.9**	****	Oxidized low density lipoprotein (lectin-like) receptor 1	NA
*Tlr1*	**3.1**	*	**2.1**	Ns	**7.5**	****	Toll-like receptor 1	NA
*C3ar1*	**2.0**	***	**3.1**	**	**7.1**	***	Complement component 3a receptor 1	Herein
*Ddx58*	**1.6**	**Ns**	**2.5**	***	**6.3**	****	DEAD (Asp-Glu-Ala-Asp) box polypeptide 58 (RIG-I)	NA
*C5ar1*	**1.4**	**Ns**	**2.0**	**	**4.5**	****	Complement component 5a receptor 1	Herein
*Adora3*	**ND**	**2.0**	**	**4.3**	**	Adenosine A3 receptor	NA
*Cd14*	2.4	**	1.9	**	**4.3**	****	CD14 Antigen	Delayed 8 days [82]
*P2ry6*	**ND**	**-**	**2.1**	Ns	**4.1**	*	Pyrimidinergic receptor P2Y, G-protein coupled, 6	NA
*Tlr8*	1.2	Ns	1.3	Ns	**4.1**	*	Toll-like receptor 8	NA
*Tlr9*	1.4	Ns	1.3	*	**3.3**	****	Toll-like receptor 9	NA
*C5ar2*	**ND**	**-**	**2.2**	*	**3.1**	**	G protein-coupled receptor 77	NA
*Tlr4*	**1.6**	**Ns**	**2.1**	***	**3.0**	***	Toll-like receptor 4	Accelerated 15–22 days [33]
*Tlr7*	2.0	*	1.5	ns	**2.8**	*	Toll-like receptor 7	NA
*Nlrp3*	-1.3	Ns	1.2	ns	**2.4**	***	NLR family, pyrin domain containing 3	No effect [83]
*Tlr6*	1.3	Ns	1.1	ns	**2.4**	*	Toll-like receptor 6	NA
*MyD88*	1.3	Ns	1.3	ns	**2.3**	***	Myeloid differentiation primary response gene 88	No effect [51]
*Tlr3*	1.8	**	-1.1	ns	**2.0**	*	Toll-like receptor 3	NA
**Unchanged**								
**Gene**	**Thalamus 60 dpi**		**Brain 94 dpi**		**Brain 131 dpi**		**Description**	**Effect of loss on prion disease**
	**FC**	**P value**	**FC**	**P value**	**FC**	**P value**		
*Adora1*	ND	-	1.1	ns	-1.1	ns	Adenosine A1 receptor	NA
*Adora2a*	ND	-	-1.1	ns	-1.2	ns	Adenosine A2a receptor	NA
*Adora2b*	ND	-	1.1	ns	-1.1	ns	Adenosine A2b receptor	NA
*Nod1*	-1.5	ns	1.2	ns	1.6	ns	Nucleotide-binding oligomerization domain containing 1	NA
*Nod2*	-1.6	ns	1.6	ns	1.7	ns	Nucleotide-binding oligomerization domain containing 2	NA
*Tlr5*	-1.3	ns	1.6	ns	1.1	ns	Toll-like receptor 5	NA
*P2ry2*	ND	-	1.6	ns	1.2	ns	Purinergic receptor P2Y, G-protein coupled 2	NA
*P2ry4*	ND	-	-1.0	ns	2.0	ns	Pyrimidinergic receptor P2Y, G-protein coupled, 4	NA
*P2ry10*	ND	-	1.3	ns	2.2	ns	Purinergic receptor P2Y, G-protein coupled 10	NA
*P2ry12*	ND	-	1.0	ns	1.4	ns	Purinergic receptor P2Y, G-protein coupled 12	NA
*P2ry13*	ND	-	1.0	ns	1.9	ns	Purinergic receptor P2Y, G-protein coupled 13	NA
*P2ry14*	ND	-	1.1	ns	1.5	ns	Purinergic receptor P2Y, G-protein coupled, 14	NA

Fold Change (FC) in 22L C57BL/10 infected mice relative to uninfected C57BL/10 mice (n = 4 for all cohorts).

P values are calculated using the student t-test

* P value ≤ 0.05

** P value ≤ 0.01

*** P value ≤ 0.001

**** P value ≤ 0.0001, ns = not significant.

ND, not determined. NA, not yet assessed during prion disease.

Underlined genes are predominantly expressed by microglia in the normal adult mouse brain [34].

### Assessment of potential ligands in the brain for targeted DAMP receptors

**Fig 1:**
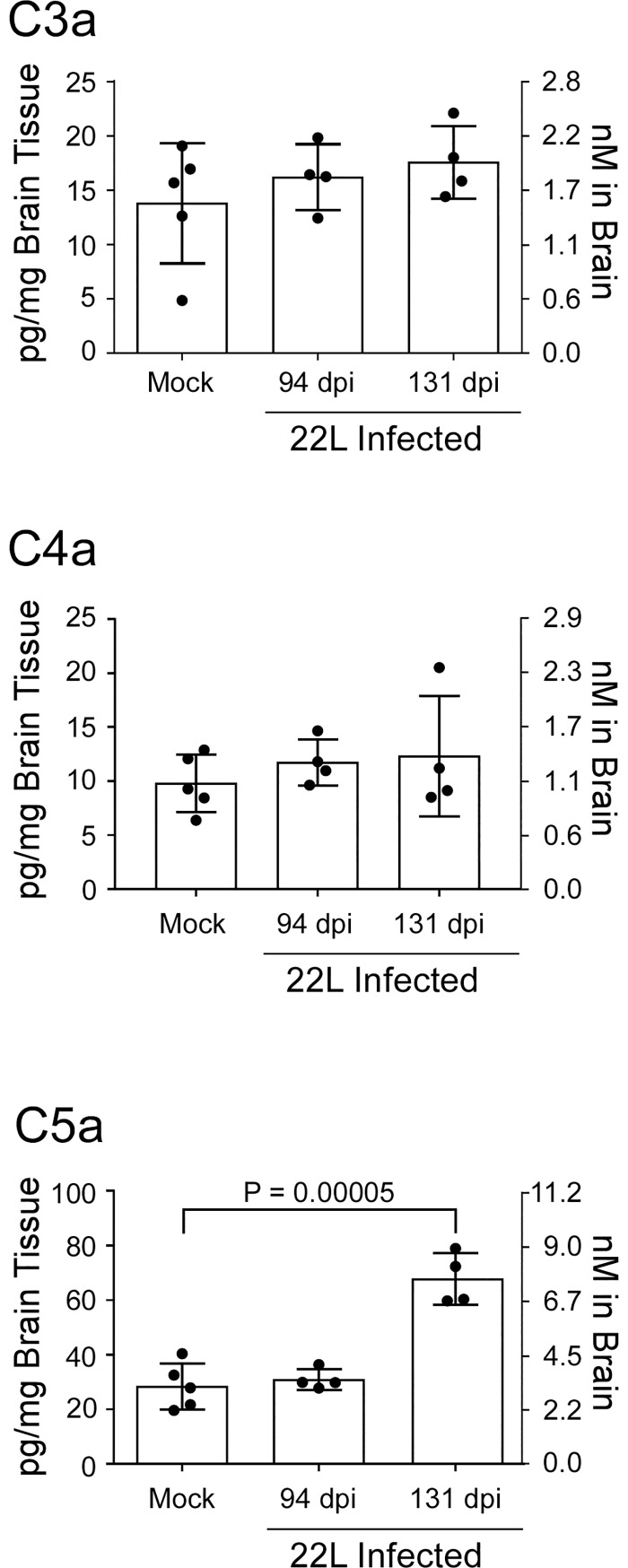
Determination of anaphylatoxin concentration in brain homogenates from 22L-infected mice. The concentration of C3a, C4a, and C5a were determined by ELISA in the brains of mice at preclinical (94 dpi) and at clinical (131 dpi) time points after scrapie infection. Mock infected brain homogenate served as a control. Scrapie-infected cohorts were compared to mock-infected controls using a one-way ANOVA.

During complement activation components C3, C4, and C5 are cleaved. The large fragments are functional proteins that are either incorporated into convertase enzymes or make up a component of the membrane attack complex. The smaller fragments C3a, C4a, and C5a are collectively referred to as anaphylatoxins, and in the case of C3a and C5a these cleavage fragments act as ligands for the innate immune receptors C3aR1 and C5aR1, respectively [43,45,46]. We assessed changes in the concentration of complement fragments C3a, C4a, and C5a in mouse brain homogenates at pre-clinical and clinical times during 22L infection (Fig 1).

The concentration of C3a or C4a did not significantly change in the brains of infected mice relative to mock. In contrast, the concentration of C5a in brain more than doubled during the course of prion disease. Although C5 is not required for prion disease [27], the production of this cleavage product might influence innate immune signaling during the disease process.

### Mice deficient in *Tlr2*, but not *C3ar1* or *C5ar1*, are more susceptible to prion disease

Though transcription of cell surface receptors such as TLR2, C3aR1, and C5aR1 are increased in brain prior to clinical onset (Table 2), their influence on prion pathogenesis has not been investigated. Furthermore, there is mounting evidence that the TLRs and complement receptors like C5aR1 and C3aR1 act synergistically to promote a heightened inflammatory response [47–49]. Though complement components C3 and C5 are not required for prion pathogenesis, the G protein-coupled receptors C3aR1 and C5aR1 might influence prion disease by interacting with other unidentified ligands to elicit a response. To assess the influence of these individual innate immune receptors on CNS prion pathogenesis, we used ic inoculation of 22L scrapie in mice deficient in TLR2 (*Tlr2*^*-/-*^), C3aR1 (*C3ar1*^*-/-*^), or C5aR1 (*C5ar1*^*-/-*^).

**Fig 2:**
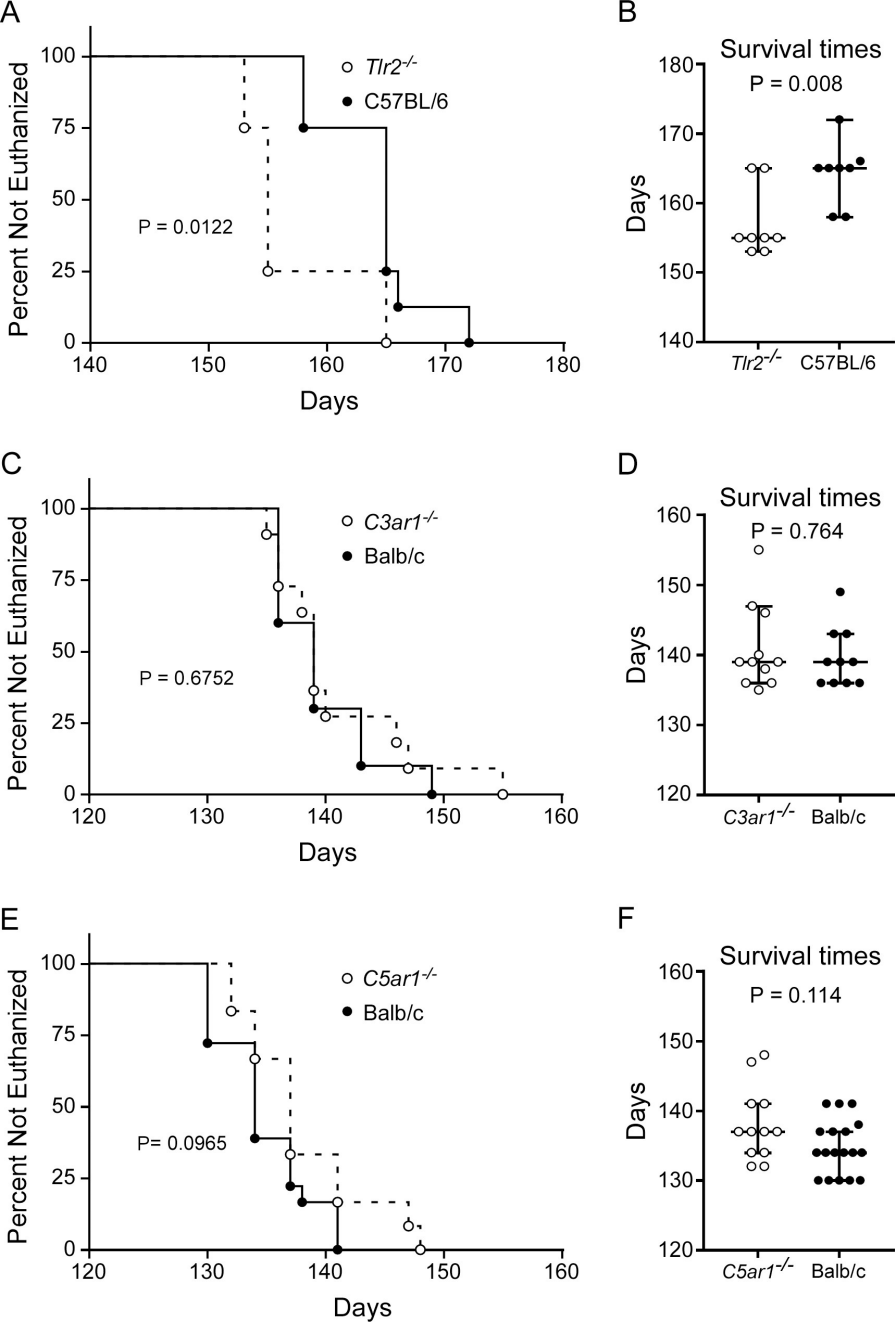
Survival of *Tlr2*^*-/-*^, *C3ar1*^*-/-*^, and *C5ar1*^*-/-*^ mice relative to control mice infected with scrapie strain 22L. Survival curves comparing 22L-infected control mice (filled circles) to mice deficient in *Tlr2* (A and B, open circles), *C3ar1* (C and D, open circles), or *C5ar1* (E and F, open circles). Infected mice were monitored by observers blinded to the genotype for clinical signs of scrapie requiring euthanasia. Data in panels A, C, and E are the percentage of animals not euthanized versus days post-infection (Days). The day each individual mouse was euthanized, the median day of euthanasia for each mouse cohort (line), and the 95% confidence interval (bars) are presented in panels B, D, and F. Mice in panels A and B were inoculated with 100-fold less 22L than *C3ar1*^*-/-*^ or *C5ar1*^*-/-*^ mice; therefore, the longer incubation periods seen with these mice were not unexpected. Statistical analysis in panels A, C, and E was performed using Mantel–Cox log-rank analysis comparing infected knockout mice to infected control mice. Statistical analysis in panels B, D, and F was performed using Mann-Whitney test comparing infected knockout mice to infected control mice.

**Fig 3:**
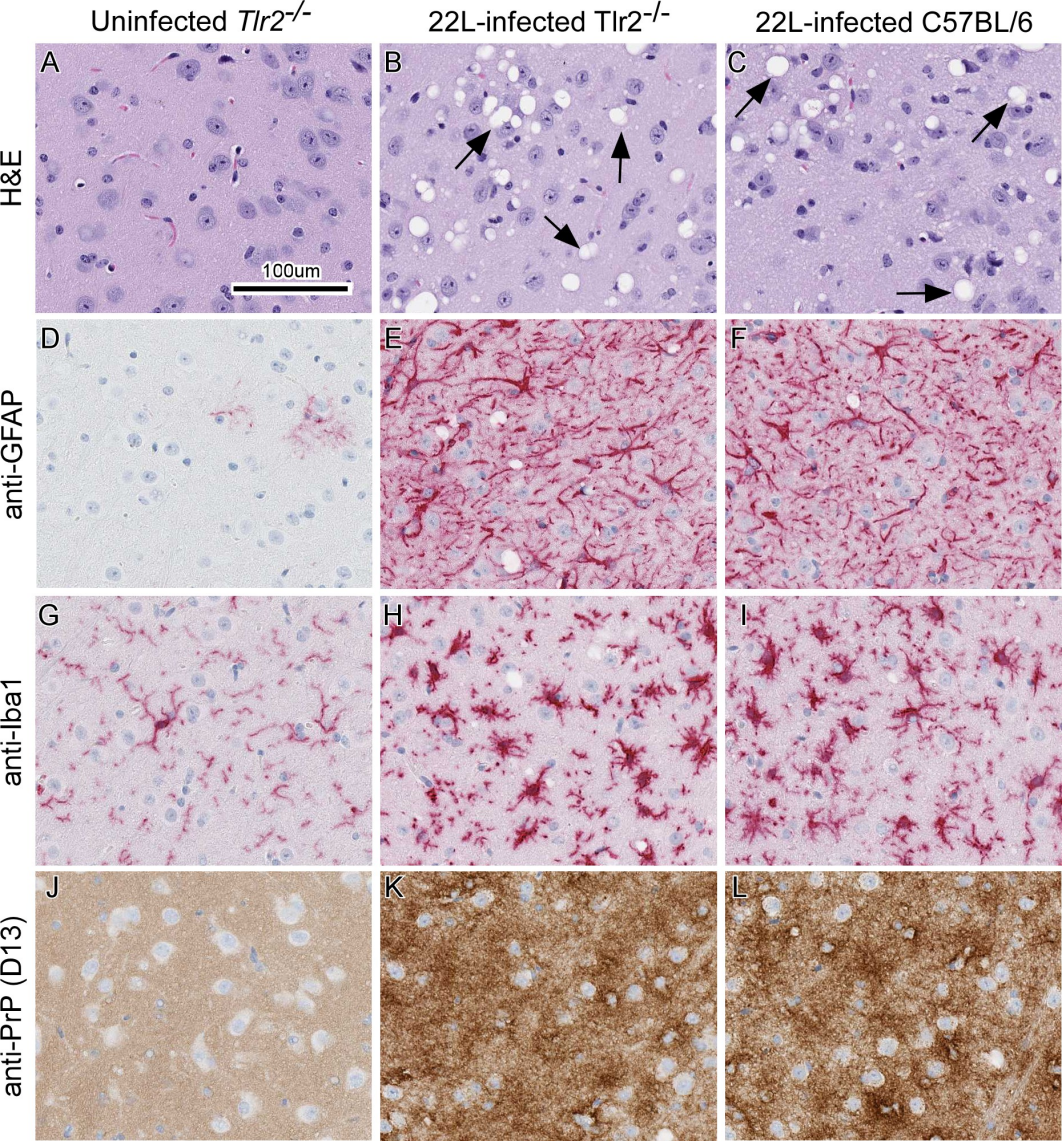
Representative neuropathology and immunohistochemical assessment of gliosis and PrP deposition in thalamic brain sections from uninfected and 22L-infected *Tlr2*^*-/-*^ mice compared to 22L-infected C57BL/6 controls. *Tlr2*^*-/-*^ mice were uninfected (panels A, D, G, and J) or infected with scrapie strain 22L (panels B, E, H, and K). For comparison C57BL/6 mice were also infected with strain 22L (panels C, F, I, and L). Sections of thalamus were stained by H&E (A-C) or probed with antibodies that recognize GFAP (D—F), Iba1 (G—I), and PrP (J—L). Representative images of the thalamus are shown for all at the same scale as indicated in panel A. Black arrows in panels B and C indicate vacuoles present in the neuropil of 22L-infected mice. Similar histopathological results were seen when 22L-infected *C3ar1*^*-/-*^ and *C5ar1*^*-/-*^ mice were compared to infected Balb/c control mice.

Absence of *Tlr2* expression resulted in a slight (median = 10 days), but statistically significant acceleration in time to euthanasia due to advanced clinical signs compared to similarly infected C57BL/6 controls (Fig 2A and 2B). This suggested that TLR2 signaling was partially protective during prion infection. Histopathological comparison of scrapie-infected *Tlr2*^*-/-*^ mice to infected control mice indicated that there was no difference in astrogliosis, microgliosis, vacuolation, or PrPSc deposition in the brains of mice that were clinical (Fig 3). Our overall results with *Tlr2*^*-/-*^ mice were analogous to previous studies using C3H/HeJ mice with impaired TLR4 signaling [33]. Together these results indicated that signaling through DAMP receptors TLR2 and TLR4, which are predominantly expressed by microglia [34], contribute to neuroprotection during scrapie infection.

Unlike *Tlr2*^*-/-*^ mice, deletion of *C3ar1* or *C5ar1* had no detectable effect on survival in 22L-infected mice relative to infected Balb/c mice used as controls (Fig 2C–2F). Infected *C3ar1*^*-/-*^, *C5ar1*^*-/-*^, and Balb/c mice were histologically indistinguishable in astrogliosis, microgliosis, vacuolation, and PrPSc deposition in the brain. Moreover, these histological observations were also indistinguishable from those of infected *Tlr2*^*-/-*^ and C57BL/6 mice in shown Fig 3. These results suggest that the loss of C3aR1 or C5aR1 signaling does not greatly influence scrapie pathogenesis.

**Fig 4:**
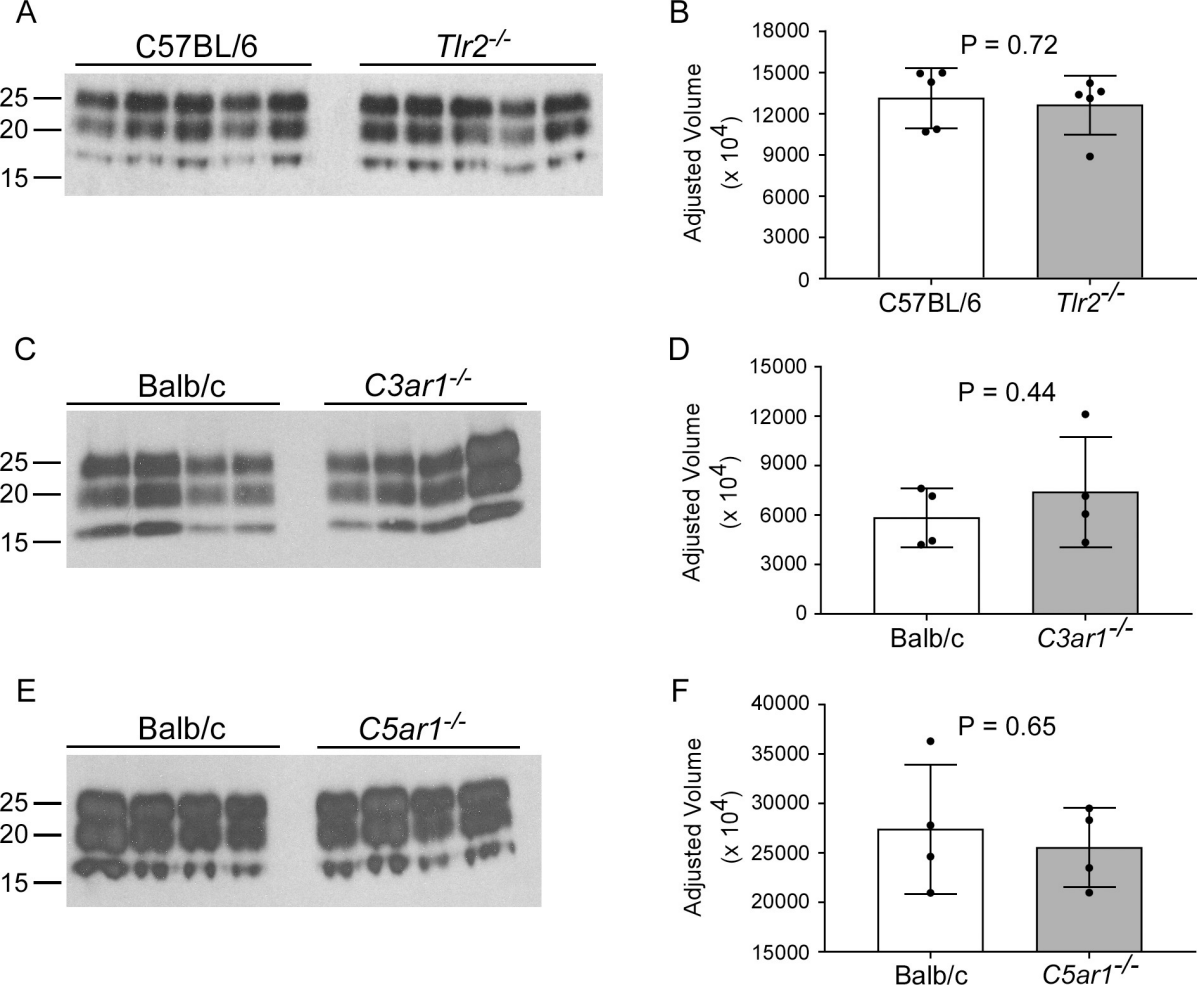
Western blot and densitometry of PrPres in the brains of knockout mice and control mice infected with 22L scrapie. *Tlr2*^*-/-*^ (A and B), *C3ar1*^*-/-*^ (C and D), and *C5ar1*^*-/-*^ (E and F) mice with appropriate control mice were infected with scrapie strain 22L and euthanized at clinical end-point. Brain homogenates were prepared and digested with proteinase K, proteins separated by SDS-PAGE, and transferred to PVDF membranes. Blocked membranes were probed with anti-PrP (D13) and bands were visualized by chemiluminescence. Panels A, C, and E were 2-minute exposures to film using ECL Western Blotting substrate. Densitometry on each lane was performed to assess differences between the groups, and plots of the adjusted volumes (see Methods) are shown to the right of the corresponding immunoblots (panels B, D, and F). Statistical analysis was performed using a two-tailed t-test comparing knockout mice to control mice. P values are as indicated.

Additional assessment of disease-associated protease resistant PrP (PrPres) accumulation was performed by immunoblot. Densitometry of immunoblots of 22L-infected mouse brain homogenates indicated PrPres accumulated to similar levels in all knockout mice relative to control mice (Fig 4). These results agreed with our histological examination of brain sections stained for PrP (Fig 3J, 3K, and 3L) and suggested that deletion of *Tlr2*, *C3ar1*, or *C5ar1* did not affect PrP deposition.

### Transcription of proinflammatory genes is largely unaffected by loss of *Tlr2* or *C5aR1* expression during prion infection

TLR2 and C5aR1 are involved with inflammatory signaling cascades that can act independently or synergistically [47–49]. Therefore, using the Mouse Inflammatory Cytokine & Receptors array (Qiagen) we assessed 84 genes associated with inflammation by qRT-PCR to quantify potential changes in proinflammatory expression during prion infection with the deletion of *Tlr2* or *C5ar1*. Only three genes were significantly altered, albeit slightly, in the brains of infected *Tlr2*^*-/-*^ mice relative to infected control mice (Table 3). Comparing 22L-infected *C5ar1*^*-/-*^ to infected control mice, the expression of only five genes was slightly increased in mutant mice (Table 4). Of note, the genes that were altered in *C5aR1*^*-/-*^ mice differed from those in the *Tlr2*^*-/-*^ mice (compare Tables 3 and 4). Furthermore, most of the pro-inflammatory genes found on this array, which we have previously identified to be increased with 22L infection (such as *Cxcl10*, *Ccl8*, *Tnf*, *IL1a*, and *IL1b*) [6,21,38,50], were similarly increased in the absence of TLR2 or C5aR1 signaling during scrapie infection. These data suggest that the majority of the ongoing inflammatory response in the CNS during prion disease is not reliant solely on signaling through DAMP receptors TLR2 or C5aR1.

**Table 3:** Proinflammatory genes altered in brains of 22L-infected *Tlr2*^*-/-*^ mice relative to 22L-infected C57BL/6 mice at the terminal timepoint.

Gene	FC	P value	Description
*Ccl2*	2.0	**	Chemokine (C-C motif) ligand 2
*Cxcl11*	-2.1	*	Chemokine (C-X-C motif) ligand 11
*IL4*	-3.2	*	Interleukin 4

Fold Change (FC) in infected *Tlr2*^*-/-*^ mice (n = 4) relative to infected C57BL/10 mice (n = 4).

P values are calculated using the Student t-test

* P value ≤ 0.05

** P value ≤ 0.01.

See Supplemental data for full analysis.

**Table 4:** Proinflammatory genes altered in brains of 22L-infected *C5ar1*^*-/-*^ mice relative to 22L-infected Balb/c mice at the terminal timepoint.

Gene	FC	P value	Description
*Ccl9*	2.2	**	Chemokine (C-C motif) ligand 9
*Ccr2*	2.3	*	Chemokine (C-C motif) receptor 2
*Csf2*	3.7	*	Colony stimulating factor 2 (granulocyte-macrophage)
*Cxcl13*	3.5	**	Chemokine (C-X-C motif) ligand 13
*Tnfsf11*	3.6	*	Tumor necrosis factor (ligand) superfamily, member 11

Fold Change (FC) in infected *C5ar1*^*-/-*^ mice (n = 4) relative to infected Balb/c mice (n = 4).

P values are calculated using the Student t-test

* P value ≤ 0.05

** P value ≤ 0.01.

See Supplemental data for full analysis.

## Discussion

We have identified numerous innate immune receptors that are increased early in the thalamus and in the brain prior to clinical signs of prion disease (Table 2). Only a few of these innate immune receptors have been studied in the context of prion infection. Thus, our aim was to assess the influence of TLR2 and the anaphylatoxin receptors, C3aR1 and C5aR1, during infection with scrapie strain 22L. Our results with *Tlr2*^*-/-*^ mice were similar to those of Spinner et al. using prion-infected C3H/HeJ mice that lack TLR4 signaling [33]. Loss of either TLR2 or TLR4 accelerates the disease. The influence of TLR2 and TLR4 in prion infection appears to be MyD88-independent because deletion of *MyD88*, an adaptor molecule often shared between TLRs, does not alter the course of prion pathogenesis [51]. Thus, the protective aspects of TLR2 and TLR4 signaling might be coordinated through a different adaptor molecule like the TIR domain-containing adaptor protein inducing IFN-β (TRIF). This is supported by experiments using mice with deletion of interferon regulatory factor 3 (*Irf3*) [52], a key transcription factor of the TRIF-dependent pathway. Prion infection of *Irf3*^*-/-*^ mice results in acceleration of disease onset [52], which is similar to our results with *Tlr2*-deficient mice and to studies performed in C3H/HeJ mice [33].

TLR2 can form a homodimer or a heterodimer with TLR1 or TLR6, increasing its repertoire of interactive ligands [53–56]. The involvement of microglial TLR2-ligand interaction in neurodegenerative diseases has become increasingly apparent. For example, TLR2 has been implicated as a primary receptor for amyloid β peptide [57], and this interaction is thought to facilitate an increase in neuroinflammation during Alzheimer’s disease. Furthermore, α-synuclein released from neurons can stimulate proinflammatory gene expression by microglia specifically through the interaction of α-synuclein and TLR2 [58,59], suggesting that TLR2 signaling may be involved in many neurodegenerative diseases including Parkinson’s disease and dementia with Lewy bodies.

To date, the ability of TLR2 to interact with PrPSc has not been addressed, but it is interesting that *Tlr2* and *Tlr1* expression was increased early in the thalamus in our study. This might suggest the formation of a TLR1/TLR2 heterodimer by microglia in response to prion infection, although we have no direct evidence for heterodimer formation at this time. It is noteworthy that mixed glia cultures upregulate *Tlr1* and *Tlr2* when exposed to recombinant prion fibrils, and preexposure of mixed neuronal and glia cultures to recombinant prion fibrils reduces prion replication in these cultures [60]. It is possible that a similar mechanism is involved during prion infection in vivo, where signaling through the TLR1/TLR2 heterodimer might reduce prion replication enough to extend survival in infected mice.

C5a is considered an extremely potent inflammatory effector that influences many cell types by increasing chemotaxis [61], augmenting vasodilation [62,63], and enhancing chemokine release [64–67]. Signaling through C5aR1 via the ligand C5a is known to contribute to neuroinflammation and pathology in neurogenerative diseases [68–71], and high concentrations of C5a can compromise the innate immune response [72–74]. The average concentration of C5a in the brain during prion infection reaches 7.6 nM by our estimate (Fig 1), and one might hypothesize that at this level C5a could contribute to the dysfunction seen in microglial phagocytosis during prion disease [75]. Surprisingly, deletion of C5, the protein that is cleaved to generate C5a, does not affect scrapie survival or vacuolation [27], and our results indicate deletion of the C5a receptor *C5ar1* also has no measurable effect on prion disease. One explanation is that elevated C5a in the brain may only play a minor role in prion pathogenesis, which is not detectable in our experiments. Alternatively, many of the other innate immune receptor-ligand systems that are likely induced during prion infection might compensate for *C5aR1* deficiency.

TLR2 and C5aR1 have been shown to act synergistically, resulting in crosstalk between the cellular pathways leading to enhanced inflammation and pathology [47–49]. For example, exposure of cultured human monocytes to LPS and C5a greatly enhances IL-6 and TNF production relative to LPS alone [76]. Considering this crosstalk and that *Tlr2*, *C5ar1*, and C5a were increased in the brain, we hypothesized that loss of either of the receptors would influence gene expression of an overlapping subset of proinflammatory markers during prion disease. Interestingly, this was not the case since the list of modestly altered proinflammatory genes from the 22L-infected *Tlr2*^*-/-*^ and similarly infected *C5ar1*^*-/-*^ mice were mutually exclusive (compare Tables 3 and 4). Furthermore, proinflammatory genes like *IL1a* and *Tnf* that are increased during prion infection [6,36,38] were similarly increased in 22L-infected *Tlr2*^*-/-*^ or *C5ar1*^*-/-*^ mice relative to infected control mice. From these experiments, there are two possible conclusions: 1) one pathway may compensate for the loss of the other, or 2) crosstalk between these pathways is unlikely in the brain during prion disease.

Various branches of the innate immune response are activated in neurodegenerative conditions including multiple sclerosis, Alzheimer’s disease, and traumatic brain injury [15,77–80]. Similarly, genes associated with complement, TLRs, and scavenging receptors are increased early and remain elevated during prion infection [4,81]. Early pathological changes in prion disease are likely due, in part, to an innate immune response to multiple DAMPs induced by damaged cells. Whether this damage is caused by direct interaction with PrPSc or by some other means is unclear. What is certain is that PrPSc associates with both neurons and astrocytes [38], and that DAMPs are potentially released by these infected or damaged cells. Activation of many of the DAMP receptors leads to a similar outcome, the translocation of NF-kB into the nucleus. Therefore, loss of any one receptor may be compensated by another intact system. Thus, it is not surprising that using any single innate immune receptor deletion mutant might yield, at best, only partial protection from prion infection. Alternative approaches such as network analysis to identify and alter “signaling bottlenecks” may be necessary to fully understand the involvement of DAMPs and the innate immune system in the brain during prion pathogenesis.

## Supporting information

S1 File (XLSX)

S2 File (XLSX)
